# Knowledge, Attitudes, and Practices of Periodic Medical Examinations Among Healthcare Professionals in a Tertiary Care Hospital in Malaysia

**DOI:** 10.7759/cureus.87459

**Published:** 2025-07-07

**Authors:** Fayiza Manzoor Ahmed, Azimatun Noor Aizuddin, Norfazilah Ahmad

**Affiliations:** 1 Department of Internal Medicine, Barking, Havering and Redbridge University Hospitals NHS Trust, Romford, GBR; 2 Department of Public Health Medicine, Faculty of Medicine, Universiti Kebangsaan Malaysia, Kuala Lumpur, MYS

**Keywords:** healthcare provider, health examination, health screen, health worker, knowledge, periodic health examination, periodic medical examination, practice

## Abstract

Background

Periodic medical examination (PME) is a fundamental step towards reducing the burden of non-communicable diseases by early detection and complication prevention. The study aimed to determine i) the knowledge, attitudes, and practices of PME among healthcare professionals in Malaysia and ii) the factors associated with the frequent practice of PME.

Methods

A cross-sectional study was conducted among healthcare professionals in a tertiary care hospital in Malaysia. A total of 317 healthcare professionals responded to the self-administered online and paper-based questionnaires.

Results

Of the respondents, 80.1% (254) had good knowledge of PME, 99.4% (315) had a positive attitude toward it, and 35.7% (113) practiced it frequently. Despite the high level of good knowledge and positive attitude, only approximately one-third of the respondents frequently practiced PME. Unmarried respondents (AOR: 0.441, 95% CI: 0.211-0.924) and those with poor knowledge (AOR: 0.462, 95% CI: 0.231-0.923) had lower odds of practicing PME regularly.

Conclusion

The burden of non-communicable diseases can be reduced by encouraging frequent practice of PME, especially among the unmarried, and improving knowledge about the importance of PME. Future national policymakers should focus on these factors to increase PME practice.

## Introduction

Non-communicable diseases have taken their toll in the modern century. The effort to reduce the burden of these diseases follows the general principle of prevention. Periodic Medical Examination (PME) is also termed as Health Screening or General Health Checkup. Screening is the identification of unrecognized disease in an apparently healthy and asymptomatic population via tests, examinations, or other procedures that can be applied rapidly and easily to the target population [1] and PME is one or more visits to a healthcare provider with the purpose of assessing overall well-being and risk factors for preventable diseases to reduce the burden of chronic diseases. A healthcare professional, also named a health worker [2], is one who provides essential services that promote health, prevent diseases, and deliver healthcare services to individuals, families, and communities based on the primary health care approach. Everyone looks at healthcare professionals as role models, and they are expected to really practice what they preach. But when it comes to keeping up to date with personal health habits and health screening practices by health professionals, there seems to be a gap in what is said and what is done by them [3]. Also, the beliefs of healthcare professionals concerning the effectiveness of different screening practices may vary by their occupation categories, as reported in a study in Brazil [4]. According to a survey conducted in the U.S., the personal health habits of healthcare workers have a great influence over their patient counseling practices [5]. Many internists often practice methods of health screening that are not recommended by experts [4].

There are international health screening guidelines available, such as those from the U.S. Preventive Services Task Force (USPSTF or Task Force), for healthcare professionals in Malaysia to follow, but no study has been done to date to know about the extent to which these guidelines are followed and how much healthcare professionals put into practice their knowledge of health screening. Also, little is known about the personal health habits of healthcare professionals. As previous literature shows the association of uptake of screening procedures with their knowledge and attitude [6], this study assessed the level of knowledge, attitude, and practice of healthcare professionals in Malaysia towards PME. The study aimed to assess the knowledge, attitudes, and practices of healthcare professionals in Malaysia regarding PME and the factors associated with its practice.

## Materials and methods

The location of this study was Hospital Canselor Tuanku Muhriz (HCTM), also known as Universiti Kebangsaan Malaysia Medical Centre (UKMMC). A cross-sectional study was done from April 2019 to January 2020, involving the healthcare professionals currently employed at HCTM. Professionals who were permanent employees of HCTM and had been working for more than one year at HCTM were included in the study. Simple random sampling was employed in this study by obtaining the list of all healthcare professionals currently working at HCTM and generating a random list using SPSS v. 23.0 (IBM Corp., Armonk, NY, US). With the help of the hospital management of HCTM, the questionnaire was sent to selected employees through emails containing the link to the Google Form. The response rate of respondents from the Google Form was 10.22%. Therefore, to achieve a minimal sample size, hardcopy questionnaires were distributed to all healthcare professionals in random departments of HCTM, ensuring the exclusion of those who had already responded to the online questionnaire. The response rate from the paper-based questionnaire was 100%.

The sample size was calculated based on the prevalence of good knowledge among healthcare workers obtained from a previous study in Saudi Arabia [7]. A self-administered questionnaire, developed by the researchers based on past literature and adopted from a study in Vietnam [8], was distributed through Google Forms and a paper-based questionnaire. The questionnaire had four sections: Section A was about the sociodemography of healthcare professionals; Section B covered questions about their knowledge of periodic medical examinations; Section C assessed their attitude toward PME; and Section D focused on their practice of PME. The questionnaire was in English and was not translated into any other language. It was validated by experts for content validity, and a pre-test study was done to check its construct validity. A reliability test was performed, and Cronbach's Alpha value was obtained (0.813 for knowledge and 0.837 for attitude) to ensure that the internal reliability of the questionnaire was good. Questions were analyzed for consistency, and questions with a Cronbach's Alpha value that was too low were removed to enhance overall reliability. Data were collected from April 2019 to September 2019. Respondents were asked six questions on knowledge. Two options were provided for the answer (Yes and No). The correct answer was given 1 mark. A good or poor level of knowledge was determined based on the mean knowledge score. For analysis, the respondents having a score of ≥4 were labeled as having good knowledge, and those having a score of <4 had poor knowledge. The respondent’s attitude was measured by 10 questions on a 5-point Likert scale. This section contained 10 questions, and participants were required to choose the option that most represented their opinion on health screening. The total possible marks for this section ranged from 10 to 50 marks. Results were presented as mean scores and in categories of positive and negative attitudes. For analysis, a score of ≥25 was considered a positive attitude, and a score of <25 was marked as a negative attitude. Frequent practice of PME is defined as health screening done in the past 12 months, whereas those who did not have health screening done in the past year were considered non-frequent practice. For analysis, those who had positive answers to both the questions asked for practice (score=2) were regarded as having frequent medical checkups, and those with a score of <2 were considered non-frequent practice. Education level was defined as the highest level of academic qualification obtained. The initial categories in the questionnaire were Diploma, Degree, Master's, and PhD. At the time of analysis, the level of education was divided into the lowest level of education (Diploma, Degree) and the highest level of education (Master's, PhD).

Statistical analysis

The data were analyzed statistically using SPSS v. 23.0. Frequency and percentage, and median and interquartile range were used to determine the objectives on knowledge, attitudes, and practices. The study variables were then categorized to determine the association between sociodemographic, socioeconomic, and lifestyle factors, bivariate analysis was done using Pearson’s chi-squared test. Also, multivariate analysis was done using binary logistic regression.

## Results

Out of a total of 2,123 healthcare professionals employed at HCTM, 221 filled out the Google Forms questionnaire. To achieve the minimum sample size, another 150 paper-based copies of the questionnaire were distributed. After excluding the missing and incomplete responses, 96 were included in the analysis. Thus, the response rate of this study for Google Forms was 10.4% and for the paper-based questionnaire, it was 80%. The median age of respondents was 34 years, and the median total personal monthly income was RM 4,800. The majority of the respondents were female (73.2%), and 88.6% were Malay by race. Most of the respondents had education up to diploma (37.2%) and degree levels (37.8%). Almost 80% of the respondents were married, and nearly one-third were doctors by profession. Only 3.2% were smokers, only 6 of the respondents could be termed as drinkers, and the majority (77%) of the respondents were physically inactive.

Table 1 shows the level of knowledge, attitudes, and practices regarding PME and their distribution on the basis of categories of healthcare professionals. Among them, doctors had the most adequate knowledge of PME (92.7%), followed by pharmacists (78.6%), allied health (74.7%), and, lastly, nurses (71.7%). Almost all (99.4%) of the healthcare professionals had a positive attitude towards PME, except a few doctors (0.6%). About two-thirds of the respondents had a non-frequent practice of PME, and the frequency of practice was better for doctors (44%) as compared to non-doctors (pharmacists=35.7%, nurses 32.3%, and allied health=29.5%).

**Table 1:** Knowledge, attitude, and practice status of respondents

Variable	Score	Total N (%)	Doctors (n=109)	Pharmacists (n=14)	Nurses (n=99)	Allied Health (n=95)
Knowledge	Good (≥4)	254 (80.1)	101 (92.7%)	11 (78.6%)	71 (71.7%)	71 (74.7%)
	Poor (≤3)	63 (19.9)	08 (7.3%)	03 (21.4%)	28 (28.3%)	24 (25.3%)
Attitude	Positive (≥26)	315 (99.4)	107 (98.2%)	14 (100%)	99 (100%)	95 (100%)
	Negative (≤25)	02 (0.6)	02 (1.8%)	00 (0.0%)	00 (0.0%)	00 (0.0%)
Practice	Frequent (=2)	113 (35.7)	48 (44.0%)	05 (35.7%)	32 (32.3%)	28 (29.5%)
	Non-Frequent (≤1)	204 (64.3)	61 (56.0%)	09 (64.3%)	67 (67.7%)	67 (70.5%)

The association of the significant sociodemographic, socioeconomic, and personal health factors with the participants’ knowledge is shown in Table 2, and with the attitude and practice of PME in Table 3 and Table 4, respectively. The education level, occupation, and total personal income per month of the participants had a significant association with their knowledge of PME. A significant association was also seen between the practice and knowledge of PME. Those with no medical history had a more positive attitude than those with a medical history of illness. Participants aged 40 years and above (48.8%) and ever-married participants (39.7%) had more frequent practice. Lastly, doctors (44.0%) and those with a monthly income of more than RM5000 (42.9%) had a more frequent practice of PME.

**Table 2:** Bivariate analysis of factors significantly associated with knowledge of participants Pearson’s chi-squared test was performed, with the level of significance set at p <0.05.

Variable	Good Knowledge=254 (80.1%)	Poor Knowledge=63 (19.9%)	Chi-square	p-value
Education				
Lowest level of education	184 (77.3%)	54 (22.7%)	4.753	0.029
Highest level of education	70 (88.6%)	09 (11.4%)	NA	NA
Occupation				
Doctors	101 (92.7%)	08 (7.3%)	16.39	<0.001
Non-doctors	153 (73.6%)	55 (26.4%)	NA	NA
Total personal income per month (RM)				
< RM 5000	121 (75.2%)	40 (24.8%)	5.077	0.024
> RM 5000	133 (85.3%)	23 (14.7%)	NA	NA
Practice				
Frequent (=2)	100 (88.5%)	13 (11.5%)	7.724	0.005
Non-frequent (≤1)	154 (75.5%)	50 (24.5%)	NA	NA

**Table 3:** Bivariate analysis of factors associated with the attitudes of participants Pearson’s Chi-squared test was performed. ^a^Yates correction was applied. The level of significance was set at p <0.05.

Variable	Positive Attitude=315 (99.4%)	Negative Attitude=02 (0.6%)	Chi-square	p-value
Gender				
Male	85 (100.0%)	00 (0.0%)	0.003^a^	0.954
Female	230 (99.1%)	02 (0.9%)	NA	NA
Age				
20-39	233 (100.0%)	00 (0.0%)	2.431^a^	0.119
40 and above	82 (97.6%)	02 (2.4%)	NA	NA
Ethnicity				
Malay	279 (99.3%)	02 (0.7%)	0.000^a^	1
Non-Malay	36 (100.0%)	00 (0.0%)	NA	NA
Marital Status				
Ever-Married	256 (99.6%)	01 (0.4%)	0.048^a^	0.826
Unmarried	59 (98.3%)	01 (1.7%)	NA	NA
Education				
Lowest level of education	237 (99.6%)	01 (0.4%)	0.000^a^	0.998
Highest level of education	78 (98.7%)	01 (1.3%)	NA	NA
Occupation				
Doctors	208 (100.0%)	00 (0.0%)	1.472^a^	0.225
Non-Doctors	107 (98.2%)	02 (1.8%)	NA	NA
Total income per month (RM)				
< RM 5000	160 (99.4%)	01 (0.6%)	0.000^a^	1
> RM 5000	155 (99.4%)	01 (0.6%)	NA	NA
Medical History				
Yes	59 (96.7%)	02 (3.3%)	8.447	0.004
No	256 (100.0%)	00 (0.0%)	NA	NA

**Table 4:** Bivariate analysis of factors significantly associated with the practice of participants Pearson’s chi-squared test was performed. The level of significance was set at p <0.05

Variable	Frequent Practice=113 (35.7%)	Non-Frequent Practice=204 (64.3%)	Chi-square	p-value
Age				
18-39	72 (30.9%)	161 (69.1%)	8.632	0.003
40 and above	41 (48.8%)	43 (51.2%)	NA	NA
Marital Status				
Ever-Married	102 (39.7%)	155 (60.3%)	9.671	0.002
Unmarried	11 (18.3%)	49 (81.7%)	NA	NA
Occupation				
Doctors	48 (44.0%)	61 (56.0%)	5.098	0.024
Non-doctors	65 (31.3%)	143 (68.8%)	NA	NA
Total Personal Income per Month (RM)				
< RM 5000	46 (28.6%)	115 (71.4%)	7.139	0.008
> RM 5000	67 (42.9%)	89 (57.1%)	NA	NA
Medical History				
Yes	29 (47.5%)	32 (52.5%)	4.658	0.031
No	84 (32.8%)	172 (67.2%)	NA	NA
Knowledge				
Good (≥4)	100 (39.4%)	154 (60.6%)	7.724	0.005
Poor (≤3)	13 (20.6%)	50 (79.4%)	NA	NA

From multivariate analysis, factors associated with frequent practice were marital status and knowledge of the PME of the participants. The most important contributing factor for non-frequent practice was poor knowledge of PME, with an adjusted odds ratio of 0.46, as shown in Table 5.

**Table 5:** Multivariate analysis of significant factors affecting the practice of participants A simple logistic regression test was done. The level of significance was p<0.05. Adj. OR = adjusted odds ratio

Variable	B	S.E.	Wald	df	p-value	Adj. OR	95% C.I for Adj. OR	
							Lower	Upper
Age	0.036	0.020	3.321	1	0.068	1.036	0.997	1.077
Income	0.000	0.000	0.143	1	0.705	1.000	1.000	1.000
Medical history	-0.315	0.312	1.023	1	0.312	0.730	0.396	1.344
Marital status	-0.815	0.377	4.672	1	0.031	0.443	0.211	0.927
Knowledge	-0.782	0.355	4.860	1	0.027	0.458	0.228	0.917
Occupation	0.259	0.298	0.754	1	0.385	1.296	0.722	2.325
Education	0.126	0.350	0.129	1	0.720	1.134	0.571	2.253

## Discussion

This study explored the knowledge, attitudes, and practices of healthcare professionals in Malaysia regarding PME and its association with their different characteristics. The majority of the respondents in this study had good knowledge of PME, similar to a study done in Taiwan [9]. This is what is expected from medical personnel: to have a higher knowledge level than the general population. Also, the majority of the respondents knew that not only those beyond age 40 need to receive regular health examinations. However, this overall percentage was less than that of the medical students in Taiwan [9]. This may be due to the variety of respondents in this study, as this study included various categories of health professionals and the level of knowledge also varied among them.

Almost all respondents in this study had a positive attitude toward periodic medical examinations. The respondents had a positive view of all 10 questions asked, in contrast to the study in Taiwan, where only half of the respondents held positive views on perspective-related questions [9]. This could be due to the difference in the level of education and understanding between medical students and healthcare professionals. Only a few of the respondents said they would go to a health check-up at the request of their employer/insurance agent. This is in contrast to a study done among hospital workers in Nigeria [10].

In this study, roughly one-third of those who had ever had a medical examination did so on a regular basis, compared to half of the respondents in the Nigerian study [10]. The reason for this could be that this study was conducted in Malaysian healthcare settings. They have a busy schedule due to long working hours and less time to get medical examinations frequently, while the study in Nigeria was done in a community setting.

PME appears to be taken up inequitably with gender, age, and socio-demographic and socioeconomic status. No significant association was found between gender and knowledge of periodic medical examinations, as well as attitude toward it, in this study, in contrast to previous studies [11-12]. In terms of practice, females had more frequent practice of this examination as compared to males. Attenders, in general, were older than non-attenders in this study, similar to many other studies [13,14]. Participants who were ever-married had a more frequent PME than single participants, as reported by other researchers [15-17]. This shows the importance of marriage and relationships where a partner plays a role in encouraging a person to self-care and regular health checkups.

The results of this study also showed that those with a lower level of education were mostly non-attenders, similar to another study [18]. This is to be expected, as those with a higher level of education are more likely to seek health care. As a result, communities with low levels of education and income should be targeted for health education, screening promotion, and disease prevention. No significant association was found between personal health habits and frequent practice of PME as stated by Ilesanmi et al. [12]. Lastly, the knowledge level of participants regarding PME in this study had a significant association with practice. This finding shows that more effort is needed to ensure that the target population receives adequate and accurate knowledge of the purpose, frequency, and place to get periodic health checkups.

Limitations and recommendations

Response bias could be a possibility since respondents who agreed to participate in the study were mostly health-conscious people who were willing to participate in health-related studies. This can be considered as one of the study limitations, as is the cross-sectional nature of this study. Furthermore, it was done among healthcare professionals of only one public, tertiary-care hospital in Malaysia, so the results have to be used cautiously for the whole population. For future research, in-depth interviews with healthcare professionals can lead to a better understanding of their uptake of regular health examinations.

Practical implications

In order to set an example for the general public to follow, it will be beneficial if healthcare professionals use their good knowledge and positive attitude toward PMEs by practicing them more frequently and on time. The Malaysian Ministry of Health has the authority to suggest a set of rules for preventive screening programs and their eligibility criteria. Establishing a national PME policy is a great approach to reduce morbidity and death rates and enable the early detection of chronic diseases.

## Conclusions

Periodic medical examinations serve an important role in the early detection, timely intervention, and prevention of possible medical conditions. Despite the high level of good knowledge and positive attitude, only approximately one-third of the respondents frequently practiced PME. The burden of non-communicable diseases can be reduced by encouraging frequent practice of PME, especially among the unmarried, and improving knowledge about the importance of PME. Future national policymakers should focus on these factors to increase PME practice.

## Appendices

Questionnaire on the knowledge, attitudes, and practices of healthcare professionals

A)  Respondent’s Profile:

Gender:       ○ Male             ○ Female

Age: __________ years

Race:   ○ Malay                                            ○ Indian

            ○  Chinese                                        ○ Others

If others, please specify: ___________________

Marital Status:

○  Married

○  Divorced

○  Single

○  Widowed

Occupation:    ______________________

Education:

○  Diploma

○  Masters

○  Degree

○  PhD

Total income per month: RM  _________________

Have you ever used any form of tobacco (cigarettes, pipes, cigars, smokeless tobacco)? YES/NO

How old were you when you first started to smoke tobacco?

During your entire life, have you smoked at least 100 cigarettes? YES/NO Do you smoke tobacco now? YES/NO

○  Everyday

○  Some days

○  Not at all

How many cigarettes on average per day do you smoke?

In the PAST YEAR, how often did you drink any type of alcoholic beverage?

In the PAST YEAR, on those days that you drank alcoholic beverages, on average, how many drinks did you have?

In the PAST YEAR, on how many DAYS did you have 5 or more drinks a day of any alcoholic beverage?

Do you do regular vigorous physical activity/exercise? YES/NO

How often do you do VIGOROUS activities for AT LEAST 10 MINUTES that cause HEAVY sweating or LARGE increases in breathing or heart rate?

About how long do you do these vigorous activities each time? minutes?

Any medical illness? If yes, please specify:                                 

Family history of medical illness:

○  Diabetes Mellitus                          ○ Kidney Disease                                  ○ Stroke

○  Hypertension                                ○ Ischemic Heart Disease                    ○ COPD

○  Dyslipidemia                                 ○ Osteoarthritis/Osteoporosis             ○ Cancer

○  Heart failure                                  ○ Gout                                                   ○ Others

B)  Questionnaire on Healthcare Professionals' Knowledge

*Direction: Please choose one answer.

1)  Periodic medical check-ups can be done during illness?

○  YES          ○ NO

2)  Only those beyond age 40 need to receive periodic medical check-ups?

○  YES           ○ NO

3)  If results are normal for this check-up, no subsequent examination is necessary?

○  YES           ○ NO

4)  Pre-employment medical check-up is a type of periodic medical check-up?

○  YES            ○ NO

5)  Health examinations are only provided by major hospitals?

○  YES            ○ NO

6)  Periodic medical check-ups should be done annually?

○  YES            ○ NO

C)  Questionnaire on Healthcare Professionals’ Attitudes

*Direction: Please read each statement carefully and circle one answer. (1=extremely disagree, 2=disagree, 3=neutral, 4=agree, 5=extremely agree).

1)  Attending periodic health check-ups is very time-consuming.

1

2

3

4

5

2)  Periodic health check-ups are necessary for the maintenance of good health.

1

2

3

4

5

3)  I will not attend periodic health check-ups because I am afraid of the results.

1

2

3

4

5

4)  I will attend periodic health check-ups because health is the first priority in life.

1

2

3

4

5

5)  I will not attend periodic health check-ups because I think my overall health is good.

1

2

3

4

5

6)  If I worry about any symptoms of ill-health, I will first seek advice from my family/friends.

1

2

3

4

5

7)  I think periodic health check-ups of employees are the responsibility of the Hospital Management.

1

2

3

4

5

8)  I will only attend periodic health check-ups at the request of my employer/insurance agent.

1

2

3

4

5

9)  The cost of periodic health check-ups is more than their long-term benefits.

1

2

3

4

5

10)  Periodic health check-ups should be made compulsory for everyone.

1

2

3

4

5

D)  Questionnaire on Healthcare Professionals’ Practices

*Direction: Please choose one answer.

1)  Have you ever done any medical examination? Yes/No. If yes, when was your latest health examination conducted?

○  <12 months

○  12-24 months

○  >24 months

○  Don’t remember

2)  Do you do health examinations annually? Yes/No

If yes, when was your latest periodic health examination?

○  <12 months

○  12-24 months

○  >24 months

○  Don’t remember

## References

[1] (2019). World Health Organization. Screening. https://www.who.int/europe/teams/ncd-management/screening.

[2] (2022). World Health Organization. Occupational Health: health workers. https://www.who.int/news-room/fact-sheets/detail/occupational-health--health-workers.

[3] Rossi TC, Bruno VHT, Catarucci FM, Beteto IdS, Habimorad PHL, Patrício KP (2019). Guidance on healthy eating habits from the medical students' perspective. Rev Bras Educ Med.

[4] Gonçalves-Silva AC, Murta-Nascimento C, Eluf-Neto J (2010). Assessing screening practices among health care workers at a tertiary-care hospital in Sao Paulo, Brazil. Clinics (Sao Paulo).

[5] Schwartz JS, Lewis CE, Clancy C, Kinosian MS, Radany MH, Koplan JP (1991). Internists' practices in health promotion and disease prevention. A survey. Ann Intern Med.

[6] Rajah R, Hassali MA, Lim CJ (2017). Health literacy-related knowledge, attitude, and perceived barriers: a cross-sectional study among physicians, pharmacists, and nurses in public hospitals of Penang, Malaysia. Front Public Health.

[7] Sait KH (2011). Knowledge, attitudes, and practices regarding cervical cancer screening among physicians in the Western Region of Saudi Arabia. Saudi Med J.

[8] Vuong QH (2016). Health communication, information technology and the public's attitude toward periodic general health examinations. F1000Res.

[9] Wu LT, Lai TY, Liu CS, Lee CC, Lin CC, Horng ML (2014). Medical students' awareness and perception of national health examinations. Biomedicine (Taipei).

[10] Akande TM, Salaudeen A (2004). Practice of periodic medical examination among hospital workers in a Nigeria teaching hospital. Niger Postgrad Med J.

[11] Cherrington A, Corbie-Smith G, Pathman DE (2007). Do adults who believe in periodic health examinations receive more clinical preventive services?. Prev Med.

[12] Ilesanmi O, Omotoso B, Alele F, Amenkhienan I (2015). Periodic medical check-up: knowledge and practice in a community in South West Nigeria. Int J Public Health Res.

[13] Bjerregaard AL, Maindal HT, Bruun NH, Sandbæk A (2017). Patterns of attendance to health checks in a municipality setting: the Danish 'Check Your Health Preventive Program'. Prev Med Rep.

[14] Zavras D, Tsiantou V, Pavi E, Mylona K, Kyriopoulos J (2013). Impact of economic crisis and other demographic and socio-economic factors on self-rated health in Greece. Eur J Public Health.

[15] Culica D, Rohrer J, Ward M, Hilsenrath P, Pomrehn P (2002). Medical checkups: who does not get them?. Am J Public Health.

[16] Hoebel J, Starker A, Jordan S, Richter M, Lampert T (2014). Determinants of health check attendance in adults: findings from the cross-sectional German Health Update (GEDA) study. BMC Public Health.

[17] Vuong QH (2017). Survey data on Vietnamese propensity to attend periodic general health examinations. Sci Data.

[18] Hsu HY, Gallinagh R (2001). The relationships between health beliefs and utilization of free health examinations in older people living in a community setting in Taiwan. J Adv Nurs.

